# Biopsychosocial Factors Associated with Supportive Care Needs in Canadian Adolescent and Young Adult Cancer Survivors

**DOI:** 10.3390/jcm10122628

**Published:** 2021-06-15

**Authors:** Steven Guirguis, Margaret Fitch, Manjula Maganti, Abha A. Gupta, Norma D’Agostino, Chana Korenblum, Jennifer M. Jones

**Affiliations:** 1 Cancer Rehabilitation and Survivorship, Princess Margaret Cancer Centre, Toronto, ON M5G 2C4, Canada; steven.guirguis@uhnresearch.ca; 2 Bloomberg Faculty of Nursing, University of Toronto, Toronto, ON M5T 1P8, Canada; marg.i.fitch@gmail.com; 3 Department of Biostatistics, Princess Margaret Cancer Centre, Toronto, ON M5G 2M9, Canada; manjula.maganti@uhn.ca; 4 Division of Hematology/Oncology, SickKids Hospital Toronto, Ontario, ON M5G 1X8, Canada; abha.gupta@uhn.ca; 5 Adolescent & Young Adult Program, Princess Margaret Cancer Centre, Toronto, ON M5G 2M9, Canada; 6 Department of Supportive Care, Princess Margaret Cancer Centre, Toronto, ON M5G 2M9, Canadachana.korenblum@sickkids.ca (C.K.); 7 Division of Adolescent Medicine, Department of Paediatrics, SickKids Hospital, Toronto, ON M5G 1X8, Canada

**Keywords:** cancer survivorship, adolescent and young adults, unmet needs, biopsychosocial factors

## Abstract

Adolescents and young adults (AYAs) represent an overlooked population in cancer survivorship care. Identifying the needs of AYAs can guide the development of tailored programs for this population. We conducted a cross-sectional descriptive analysis to identify biopsychosocial factors associated with AYA post-treatment supportive care needs and unmet needs using data obtained from the Experiences of Cancer Patients in Transitions Study of the Canadian Partnership Against Cancer, in collaboration with cancer agencies in the 10 Canadian provinces. The analysis focused on data from *n* = 530 AYAs between the ages of 18 and 34 who had undergone treatment within the past 5 years. Respondents reported a median of two moderate to big (MTB) physical needs (out of 9) and one unmet physical need, two MTB emotional needs (out of 6) with two unmet MTB emotional needs, and one (out of 5) practical need reported and one unmet MTB practical need. We found some common associations across supportive care domains. Income (lower) and more complex treatment were associated with high needs and unmet needs across the three domains. Respondents with a family doctor who was “very involved” in their cancer care had a lower number of unmet physical and emotional needs. Identifying those at risk of supportive care needs and developing tailored pathways in which they are proactively connected with tailored and appropriate resources and programs may help to reduce the number of unmet needs and improve cancer survivors’ quality of life.

## 1. Introduction

While cancer remains the leading disease-related cause of death in adolescents and young adults (AYA; defined as ages 15–39) [1,2,3], the overall 5-year survival rate has improved to over 80% [4], leading to a growing number of long-term AYA cancer survivors. This has resulted in calls for more attention to be given to the ongoing supportive care needs of this unique population and the development of tailored programs to address these needs.

AYA cancer survivors’ experiences are complex and unique compared to those of younger children and older adults [5,6,7]. While the incidence of specific cancer diagnosis can vary considerably across the AYA age continuum, the most common diagnoses in AYA populations are testicular cancer (male), breast and cervix cancer (female), thyroid cancer, non-Hodgkin lymphoma, colorectal cancer, and melanoma [8]. The diagnosis and treatment of cancer during early adulthood are variable but can result in physical and psychosocial side effects that may persist after the treatment ends and pose a life-long risk for the development of late adverse effects [6,9,10,11]. These can negatively impact AYA cancer survivors, delay their achievement of important life milestones, and present unique challenges to restoring and sustaining their health and overall wellbeing [12,13]. Undergoing cancer treatment at this age can negatively impact identity development [7,13], including the ability to develop autonomy and build intimate and emotional relationships [7,10,13]. Furthermore, undergoing treatment at this age interferes with ongoing education or employment opportunities [7,10,13]. AYAs also experience different psychological issues compared to other age groups. AYAs with cancer are significantly more distressed than older adults [14,15] and more likely to suffer from depression and anxiety [16]. Adult cancer centers, where almost all AYA survivors ≥18 years receive treatment and follow-up care, have limited expertise to address the specific care needs of this patient population [12], and a significant proportion of AYA cancer survivors do not fully engage in survivorship care and do not complete their follow-ups [17].

Despite calls for the development of specialized follow-up care and survivorship programs for this unique population [18,19,20,21], many AYA survivorship issues continue to be poorly managed [18], leading to unmet supportive care needs and concerns. Research on AYA cancer survivors remains underrepresented in the literature [18], and there is little evidence to inform the care of AYAs survivors, particularly in the follow-up survivorship period, when unmet needs are often at their highest [22]. Previous studies have reported that up to half of AYA cancer survivors report unmet informational and services needs [19,23]. These needs can be physical, emotional, or practical in nature [20]. Identifying AYA cancer survivors who have higher numbers of needs and who are at risk of not having those needs met can aid oncology teams in identifying and referring them to appropriate programs and interventions. The purpose of the current paper is to identify biopsychosocial factors associated with AYA cancer survivors’ post-treatment supportive care needs as well as unmet needs.

## 2. Methods

A cross-sectional descriptive study was conducted to analyze survey responses from a sample of AYA cancer survivors aged 18–34 years.

### 2.1. Survey Data

The surveys were acquired from a large national survey conducted by the Canadian Partnership Against Cancer (CPAC) [24]. The Experience of Cancer Patients in Transitions Study (“Transitions Study”) aimed to understand the experiences of cancer survivors 1–3 years post-treatment by identifying their needs and the factors associated with unmet needs. The survey was administered across ten Canadian provinces and was available in English and French. CPAC developed the survey after conducting literature reviews to build the conceptual framework and consulted with cancer survivors, clinicians, and system leaders to collect feedback about the framework’s relevance. Fifteen cancer survivors completed cognitive interviews and 96 survivors completed performance testing to evaluate its clarity, meaningfulness, and ease of completion as part of pilot testing. The survey, originally distributed across Canada in 2016, could be completed on paper or online. A copy of the survey is available on the CPAC System Performance site at http://www.systemperformance.ca/transition-study.

In the Transitions Study, provincial cancer registries identified eligible patients (age ≥18, 1–3 years post-treatment). All eligible survivors in smaller provinces were mailed survey packages, as they were less likely to achieve the required sample size. Provinces with a larger number of eligible survivors than required underwent random sampling for each cancer type. Out of the 40,790 surveys sent, 13,258 surveys were completed. The data from the Transitions Study were made available through CPAC and accessed in June 2019. Out of the 13,258 completed surveys, 575 respondents were AYAs between the ages of 18 and 34. Further detail regarding the survey development, sample selection, and survey dissemination is available in Fitch et al., 2018 [24], and Jones et al., 2021 [20].

The CPAC Transitions Study obtained ethics and privacy approval through each province’s respective cancer registry. The current study did not directly interact with patients or the provincial cancer registries, as the data were acquired from CPAC. All the procedures were carried out in accordance with the ethical standards of the institutional and national committee on human experimentation and with the 1964 Helsinki Declaration.

### 2.2. Description of Needs

Reported concerns and unmet needs were grouped by domain (physical, emotional, practical). Each domain covered a range of concerns. Physical concerns focused on the post-treatment physical and symptom burden. This included fatigue, fertility problems, pain, gastrointestinal problems, cognitive symptoms, and physical discomfort. Emotional concerns encompassed the AYAs’ ability to handle psychosocial demands post-treatment. These concerns covered the areas of mental health, stress, and changes in family or social relationships and ability to cope. Lastly, practical concerns covered AYAs’ need for assistance. Practical concerns focused on returning to work or school, taking care of oneself or one’s family, difficulties in getting around, and the financial costs associated with cancer treatment.

### 2.3. Statistical Analysis

The analyses focused on identifying factors associated with supportive care needs and unmet needs in AYA cancer survivors. Descriptive statistics (frequency and proportions) were calculated for demographic and clinical variables and presented according to the type of cancer experienced.

Definition of supportive care needs: Supportive care needs were defined using questions related to needs in the physical (9 questions), emotional (6 questions), and practical domains (5 questions) in the CPAC survey. Each question had a choice of answers, from no, small, moderate, or big needs or no response. For the purpose of this analysis, the response of no need or small need was coded as zero and the response of moderate or big (MTB) need was coded as one. No response was treated as missing data. Responses to the questions in each domain were summed up to count the number of needs. The number of MTB needs ranged from 0 to 9, 0 to 6, and 0 to 5 in the physical, emotional, and practical domains and were modelled as counts.

Definition of unmet needs: The denominator used for unmet needs analysis in each domain is patients who had MTB needs in that corresponding domain. Unmet need for each of the questions was coded as zero or one using the following criteria. If the respondent had an MTB need but did not seek any help, sought help for a particular need but reported that it was hard or very hard to get help, or did not get any help despite seeking it, this was coded as an unmet need with a value of one. If the respondent had an MTB need, sought help, and reported that it was easy or very easy to get help, this was coded as having no unmet need, with a value of zero. Respondents who had an MTB need but did not answer the remaining questions corresponding to unmet needs were treated as missing data. Responses for unmet needs across all questions in each domain were summed up to count the total number of unmet needs. The number of unmet needs for MTB needs ranged from 0 to 8, 0 to 6, and 0 to 5 in the physical, emotional, and practical domains and was modelled as counts.

To test the associations between the number of MTB needs; the number of unmet needs for MTB needs; and clinical, demographic, and treatment-related variables in the physical, emotional, and practical domains, univariable (UVA) and multivariable (MVA) Poisson regression analyses were employed. Whenever over-dispersion was present, the covariance matrix was adjusted by the scale parameter (option DSCALE in GENMOD procedure in SAS). Variables that had a significant association with a number of unmet needs in UVA were further tested in MVA; the associated Risk Ratios are reported along with the 95% CI. The Risk Ratio represents the fold increase in the number of needs in one group vs. another. Statistical significance was considered as *p* < 0.05. Analyses were conducted using SAS V 9.4.

## 3. Results

### 3.1. Participants

Out of the 575 completed surveys, 55 were excluded from the analyses, including patients who did not indicate that they had undergone any form of therapy for cancer (*n* = 24) and patients that indicated their cancer type as ‘Other’ (*n* = 20) or none (*n* = 1).

Demographics and clinical factors are summarized in Table 1. In total, 530 AYA responses were included in this analysis. Among these respondents, 61% identified as female. Almost half of the participants (43%) were between the ages of 30 and 34 years old, 37% were between the ages of 25 and 29 years old, and 20% were between the ages of 18 and 24 years old. Half of the sample were married or had a partner. Lastly, 60% of the responders had completed some form of post-secondary education.

### 3.2. Frequency of Moderate or Big Supportive Care Needs

**Figure 1:**
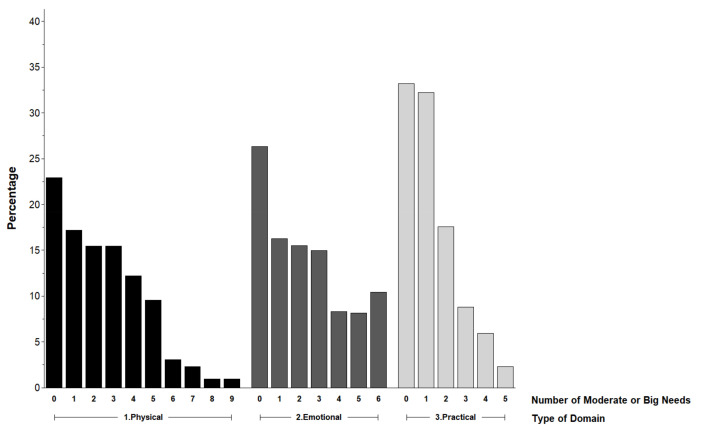
Frequency of moderate to big supportive care needs.

The frequency of the total number of MTB-rated supportive care needs within each domain is displayed in Figure 1. Respondents had a median of two physical (possible range 0–9), two emotional (possible range 0–6), and one practical needs (possible range 0–5) (Table 2). Twenty-nine percent (152/524) had ≥four physical needs, 42% (221/528) had ≥three emotional needs, and 35% (181/524) had ≥two practical needs. The frequency of needs by item and domain is reported in Supplementary Materials Table S1.

### 3.3. Frequency of Unmet Needs

**Figure 2:**
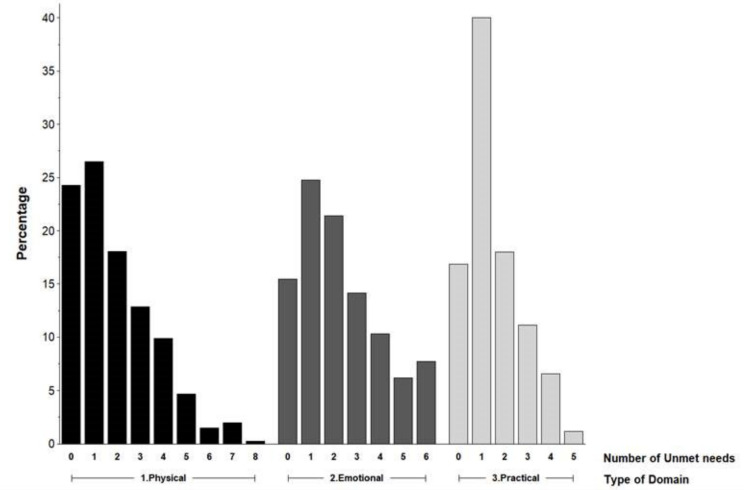
Frequency of unmet needs for moderate to big supportive care needs.

Figure 2 presents the frequency of the total number of unmet needs for supportive care needs that were rated MTB within each domain. The respondents had a median of one unmet physical, two unmet emotional, and one unmet practical needs (Table 3). Of those who reported a MTB need, 17% (66/399) had ≥four unmet physical needs, 24% (94/388) had ≥three unmet emotional needs, and 37% (181/524) had ≥two unmet practical needs.

### 3.4. Physical Domain

In the UVA, cancer type, gender, income, education, employment, and treatment type were significantly associated with the frequency of MTB physical needs (see Supplementary Materials Table S2).

Based on the final MVA (see Table 4), being female, having lower income, being unemployed or on leave from work, and having received chemotherapy were associated with a higher number of MTB physical needs.

#### Unmet Physical Needs

In the UVA for unmet physical needs, cancer type, gender, language, employment, involvement of health care personnel with follow-up care, physician type, and treatment type were significantly associated with the risk of having unmet needs for MTB physical needs (see Supplementary Materials Table S2).

The final MVA revealed that being female and having received chemotherapy had significant associations with having a higher number of MTB unmet physical needs. However, respondents with a family doctor or nurse practitioner who was “very involved” in their cancer care had lower unmet physical needs (Table 4).

### 3.5. Emotional Domain

In the UVA, cancer type, gender, chronic conditions, income, language, education, employment, and treatment type were significantly associated with the frequency of MTB emotional needs (see Supplementary Materials Table S2).

The final MVA results found that respondents who spoke English, reported having another chronic condition other than cancer, had lower income, and underwent more complex treatment regimens reported higher MTB emotional needs.

#### Unmet Emotional Needs

In the UVA, income, the population size of a geographical region, and the involvement of health care personnel with follow-up care were significantly associated with the risk of having unmet needs for MTB emotional needs (see Supplementary Materials Table S2).

Based on the final MVA (see Table 5), having a lower income and living in a rural location/town of less than 2000 people were associated with having a higher number of unmet MTB emotional needs. However, those respondents with a family doctor or nurse practitioner who was “very involved” in their cancer care had a lower number of unmet emotional needs.

### 3.6. Practical Domain

For practical needs, the UVA revealed that age group, income level, employment status, and treatment type were significantly associated with the frequency of unmet needs for MTB practical needs (see Supplementary Materials Table S2).

Based on the final MVA, being older, having a lower income, and having received certain treatments (chemotherapy, chemo/radiation, radiation) were associated with having a higher number of MTB practical needs.

#### Unmet Practical Needs

For unmet MTB practical needs, based on the UVA, age group, income, employment, and treatment type were significantly associated with the frequency of unmet practical needs (see Supplementary Materials Table S2).

Based on the final MVA (see Table 6), being older, having lower income, and having undergone more complex treatments were associated with having a higher number of MTB practical needs.

## 4. Discussion

This study explored the demographic and clinical variables associated with MTB physical, emotional, and practical supportive care needs and unmet needs in AYA (18–34 years) cancer survivors who had been diagnosed and treated for cancer. To our knowledge, this was the largest study to date examining factors associated with the frequency of MTB supportive care needs and unmet needs in AYA cancer survivors (18–34 years).

In terms of physical needs, respondents reported a median of two MTB physical needs (out of 9) and one unmet physical need. Approximately 1/3 (29%) of respondents reported four or more MTB physical needs, and 17% reported four or more unmet MTB needs. Respondents reported a median of two MTB emotional needs (out of 6) and two unmet MTB emotional needs. A substantial minority of respondents (42%) reported three or more MTB emotional needs, and almost a quarter (24%) reported three or more unmet emotional needs. Practical needs were also very common, with a median of one (out of 5) practical need reported and one unmet MTB practical need. Over a third (35%) of the respondents reported two or more MTB practical needs or unmet needs (37%). These findings are supported by the work of Zebrack et al. (2009), who found that needs for psychosocial support and counseling and practical service needs were often unmet (23).

Based on our MVA, we found some common associations across supportive care domains. Income was a significant variable across the physical, emotional, and practical domains. People who had a lower income, especially those in the lowest income bracket (<$25,000), reported the highest levels of MTB needs, as well as a higher number of unmet emotional and practical needs. Financial strain is a recognized predictor of unmet healthcare needs [22,25]. A cancer diagnosis is subject to various out-of-pocket costs (e.g., transportation to appointments) and loss of income through reduced workforce participation [26,27,28,29]. Cancer-related financial strains could trigger or intensify financial hardship and have a greater impact on low-income individuals, thus increasing the number of needs and unmet needs [30]. In addition, respondents who underwent more complex treatments compared to receiving surgery alone were also at higher risk for a higher number of MTB needs in all domains. The addition of chemotherapy and radiation therapy can result in additional and prolonged adverse effects and symptoms and could thus account for these findings. Finally, respondents with a family doctor or nurse practitioner who was “very involved” in their cancer care had lower unmet physical and emotional needs. Previous research has reported that adult cancer survivors who had both a General Practitioner (GP) and oncologist involved in their survivorship care reported a higher likelihood of met needs compared to those with GPs who were not involved in their survivorship care [31]. The collaboration between oncologists and primary care providers has been highlighted as a crucial component of high-quality survivorship care [32]. The exchange of information between care providers increases patient satisfaction and is associated with positive survivorship outcomes [33,34].

Specific to physical needs, respondents who identified as female reported having a higher number of physical MTB needs and unmet physical needs. Previous studies have shown that women reported more needs than men [35,36,37,38], and in AYA populations females report the lowest quality of life outcomes [39]. It is unclear as to whether this difference is due to women experiencing more unmet needs or other factors, such as coping and help-seeking [37,40]. This may be due to needs related to hormonal or fertility problems, which are more often reported by female AYA cancer survivors [41]. Future studies should clarify what factors lead to this difference between male and female supportive care needs. Not surprisingly, being unemployed or on leave from work was also associated with a higher number of MTB physical needs. Interventions that treat and manage physical long-term effects may help AYAs get back to work.

Emotional concerns were higher in those who reported having another chronic condition. This finding is supported by other research, which has demonstrated that cancer survivors suffering from comorbid diseases experience lower levels of health-related quality of life, including emotional function [42]. In a study of 485 AYA patients, having ≥2 comorbidities on the AYA index was associated with higher mental health service needs after adjusting for demographic and clinical factors [43]. Another interesting finding was that respondents who came from a French-speaking region had lower emotional needs compared to those who completed the survey in English. The large majority of those who completed the survey in French were from Quebec. While it is unclear why this is, the implementation of the Quebec Mental Health (MH) Reform (2005–2015), which aimed to improve the accessibility, quality, and continuity of care by developing primary care, optimizing integrated service networks [44], and promoting recovery best-practices (e.g., care pathways, cognitive behavioral therapy [45]), may have resulted in better access to mental health service, especially for those with primary care providers. Finally, in addition to lower income, those who lived in rural locations had a higher number of unmet emotional needs. This may be the result of having less access to mental health services, resulting in a higher number of unmet needs. However, as suggested by Fressen (2019), there are additional barriers that are influenced by economic, social, and cultural nuances that vary by community which require mental health programs tailored to the unique complexities of each community. These can include an increased value placed on self-reliance and the stigmatization of seeking mental health support [46].

Along with income and treatment, age was also associated with practical needs. The youngest AYA group (18–24) reported the lowest number of MTB practical needs and unmet needs. AYAs who are younger may still be living with parents and have practical and social support in place, whereas older AYAs may have more responsibilities related to finances, relationships, and families that can result in more practical needs [19]. Our finding of lower needs and unmet needs in our younger respondents is in contrast to the results of Zebrack et al. (2009) [23], who reported higher unmet information and service needs in respondents who were younger. While this difference may be attributed to different needs being assessed across the studies, the finding from both suggest that age differences exist in AYA age groups and that HCPs should be aware of this in order to better target their services.

## 5. Study Limitations

This study has a few limitations that need to be considered when interpreting the results. The overall response rate for this survey was 33%, and this may have introduced response bias. Clinical information including disease status, time since diagnosis, and treatments received was self-reported and not validated. Additionally, due to confidentiality issues regarding the characteristics of the respondents, there was insufficient detail to allow for the weighing of the surveys to ensure that they were representative of all Canadians. In terms of the survey development, while the developers did include AYA individuals during the review stage of the questionnaire design and they felt that the survey addressed the issues that concerned them, the survey was not specifically developed for AYA cancer survivors. Finally, the survey was only offered in English and French and excluded those who did not speak one of these languages.

## 6. Conclusions

This paper highlights several demographic and clinical factors that are associated with the frequency of moderate and big supportive care needs and unmet needs. These findings contribute to the current research on supportive care needs in AYA cancer survivors and may be helpful in the development of tools for the risk stratification of AYA cancer survivors in the transition to the survivorship phase. Identifying those at risk of having supportive care needs and developing tailored pathways to proactively connect survivors with tailored and appropriate resources and programs may help to reduce the number of unmet needs and improve survivors’ quality of life.

## Figures and Tables

**Table 1:** Demographics of the adolescents and young adults (AYAs) sample by cancer type (*n* = 530).

Variable	Full Sample (*n* = 530) *n* (%)	Hematologic (*n* = 141) *n* (%)	Solid Tumor (*n* = 389) *n* (%)
**Age group (years)**			
18 to 24	105 (19.81)	38 (26.95)	67 (17.22)
25 to 29	197 (37.17)	46 (32.62)	151 (38.82)
30 to 34	228 (43.02)	57 (40.43)	171 (43.96)
**Gender**			
Female	322 (61.33)	79 (56.43)	243 (63.12)
Missing	5	1	4
**Income**			
Less than $25,000	76 (14.42)	21 (15.00)	55 (14.21)
$25,000 to less than $50,000	89 (16.89)	27 (19.29)	62 (16.02)
$50,000 to less than $75,000	89 (16.89)	23 (16.43)	66 (17.05)
$75,000 to less than $125,000	120 (22.77)	32 (22.86)	88 (22.74)
$125,000 or more	85 (16.13)	17 (12.14)	68 (17.57)
Prefer not to answer	68 (12.90)	20 (14.29)	48 (12.40)
Missing	3	1	2
**Marital status**			
Single/separated/divorced/widowed	259 (48.87)	177 (45.50)	82 (58.16)
Married or partnered	271 (51.13)	212 (54.50)	59 (41.84)
**Language (survey** **completed)**			
English	304 (57.36)	65 (46.10)	239 (61.44)
French	226 (42.64)	76 (53.90)	150 (38.56)
**Education level (highest level)**			
≤High school diploma	94 (18.01)	27 (19.71)	67 (17.40)
≤College	148 (28.35)	38 (27.74)	110 (28.57)
Some university	56 (10.73)	13 (9.49)	43 (11.17)
University (Bachelors/Masters or PhD)	224 (42.91)	59 (43.07)	165 (42.86)
Missing	8	4	4
**Current employment status**			
Full time student	83 (15.75)	24 (17.14)	59 (15.25)
Full time work	283 (53.70)	62 (44.29)	221 (57.11)
Part time work	55 (10.44)	19 (13.57)	36 (9.30)
On leave/disability/not working	106 (20.11)	35 (25.00)	71 (18.35)
Missing	3	1	2
**Population size of geographic location**			
Rural/In a town <2000 people	59 (11.22)	48 (12.44)	11 (7.86)
In a town (2000 to 10,000 people)	52 (9.89)	34 (8.81)	18 (12.86)
In a small city (10,000 to 50,000 people)	99 (18.82)	72 (18.65)	27 (19.29)
In a large city (>50,000 people)	316 (60.08)	232 (60.10)	84 (60.00)
Missing	4	3	1
**Other chronic conditions**			
No	360 (67.92)	96 (68.09)	264 (67.87)
Yes	170 (32.08)	45 (31.91)	125 (32.13)
**Treatment received**			
Surgery only	188 (35.47)	1 (0.71)	187 (48.07)
Chemotherapy only	93 (17.55)	78 (55.32)	15 (3.86)
Radiation only	11 (2.08)	0 (0.00)	11 (2.83)
Chemotherapy and radiation	60 (11.32)	41 (29.08)	19 (4.88)
Surgery and chemotherapy	67 (12.64)	11 (7.80)	56 (14.40)
Surgery and radiation	41 (7.74)	2 (1.42)	39 (10.03)
Surgery, chemotherapy, and radiation	70 (13.21)	8 (5.67)	62 (15.94)
**Physician providing follow-up care**			
Oncologist	328 (62.36)	227 (58.96)	101 (71.63)
General Practitioner (GP)	49 (9.32)	45 (11.69)	4 (2.84)
GP and oncologist	128 (24.33)	94 (24.42)	34 (24.11)
No one/unsure	21 (3.99)	19 (4.94)	2 (1.42)
Missing	4	4	0
**How involved is your family doctor/general practitioner/nurse practitioner in your follow-up cancer care (combined categories)?**			
Not at all involved	101 (19.13)	67 (17.31)	34 (24.11)
Somewhat involved	254 (48.11)	191 (49.35)	63 (44.68)
Very involved	109 (20.64)	82 (21.19)	27 (19.15)
Do not have a family Doctor/GP/nurse/unsure	64 (12.12)	47 (12.14)	17 (12.06)
Missing	2	2	0

**Table 2:** Summary of moderate to big needs reported by domain.

Domain	*n*	Mean (SD)	Median	IQR	Min	Max
Physical	524	2.42 (2.07)	2	1–4	0	9
Emotional	528	2.29 (2.01)	2	0–4	0	6
Practical	524	1.29 (1.30)	1	0–2	0	5

**Table 3:** Summary of unmet needs for moderate or big supportive care needs by domain.

Type of Concern	*n*	Mean (STD)	Median	IQR	Min	Max
Physical	399	1.86 (1.67)	1	1–3	0	8
Emotional	388	2.29 (1.77)	2	1–3	0	6
Practical	350	1.48 (1.16)	1	1–2	0	5

**Table 4:** Multivariable analysis results in the physical domain.

Covariate	Moderate or Big Needs		Unmet Needs *	
	Risk Ratio (RR) (95%CI)	*p*-Value	Risk Ratio (RR) (95%CI)	*p*-Value
**Gender**				
Male	Reference	**<0.001**	Reference	**0.001**
Female	1.48 (1.25–1.76)		1.39 (1.14–1.71)	
**Income**			-	-
Less than $25,000	Reference			
$25,000 to less than $50,000	0.91 (0.71–1.18)	0.505		
$50,000 to less than $75,000	0.95 (0.73–1.23)	0.696		
$75,000 to less than $125,000	0.83 (0.64–1.07)	0.162		
$125,000 or more	0.75 (0.57–0.99)	**0.046**		
Prefer not to answer	0.69 (0.51–0.92)	**0.013**		
**Education (highest level)**			-	-
University (Bachelors/Masters or PhD)	Reference			
≤High school diploma	1.03 (0.82–1.30)	0.778		
≤College	1.23 (1.03–1.48)	**0.02**		
Some university	1.17 (0.91–1.51)	0.225		
**Current employment status**			-	-
Full Time work	Reference			
Part time work	1.08 (0.83–1.40)	0.539		
Full Time Student	1.07 (0.85–1.35)	0.552		
On leave/unemployed	1.30 (1.07–1.58)	**0.007**		
**Treatment received**				
Surgery only	Reference		Reference	
Chemotherapy only	1.87 (1.49–2.33)	**<0.001**	1.48 (1.14–1.93)	**0.004**
Radiation only	1.6 (0.96–2.65)	0.069	1.24 (0.67–2.28)	0.493
Chemotherapy and radiation	1.68 (1.29–2.19)	**<0.001**	1.24 (0.90–1.69)	0.185
Surgery and chemotherapy	1.93 (1.50–2.48)	**<0.001**	0.95 (0.68–1.32)	0.741
Surgery and radiation	1.45 (1.06–1.98)	**0.02**	1.07 (0.72–1.58)	0.735
Surgery, chemotherapy, and radiation	2.18 (1.74–2.73)	**<0.001**	1.49 (1.14–1.96)	**0.003**
**How involved is your family doctor/general practitioner/nurse practitioner in your follow-up cancer care (combined categories)?**	-	-		
Not at all involved			Reference	
Do not have a family doctor/GP/nurse/unsure			1.12 (0.82–1.53)	0.458
Somewhat involved			0.81 (0.64–1.02)	0.074
Very involved			0.66 (0.49–0.89)	**0.007**

* Unmet needs: Unmet needs for moderate to big needs. Bolded *p*-values represent significant (<0.05) variables from the multivariable analysis.

**Table 5:** Multivariable analysis results in the emotional domain.

Covariate	Moderate or Big Needs		Unmet Needs	
	Risk Ratio (RR) (95%CI)	*p*-Value	Risk Ratio (RR) (95%CI)	*p*-Value
**Gender**				
Male	Reference	**<0.001**		
Female	1.40 (1.17–1.66)			
**Income**				
Less than $25,000	Reference		Reference	
$25,000 to less than $50,000	0.95 (0.72–1.24)	0.698	0.79 (0.59–1.04)	0.093
$50,000 to less than $75,000	1.16 (0.91–1.49)	0.235	0.83 (0.64–1.08)	0.171
$75,000 to less than $125,000	0.86 (0.67–1.11)	0.254	0.78 (0.60–1.00)	0.055
$125,000 or more	0.72 (0.54–0.96)	**0.025**	0.69 (0.50–0.93)	**0.015**
Prefer not to answer	0.69 (0.50–0.95)	**0.021**	0.77 (0.56–1.05)	0.100
**Language (survey completed)**			-	-
French	Reference	**0.025**		
English	1.22 (1.02–1.44)			
**Population size of geographic location**	-	-		
Rural location/town < 2000 people			Reference	
Town (2000 to 10,000 people)			0.80 (0.57–1.14)	0.219
Small city (10,000 to 50,000 people)			0.92 (0.69–1.24)	0.590
Large city (>50,000 people)			0.74 (0.57–0.96)	**0.023**
**Other chronic condition**			-	-
No	Reference	**0.009**		
Yes	1.25 (1.06–1.48)			
**Treatment received**			-	-
Surgery only	Reference			
Chemotherapy only	1.11 (0.85–1.44)	0.436		
Radiation only	1.31 (0.76–2.28)	0.322		
Chemotherapy and radiation	1.23 (0.93–1.64)	0.151		
Surgery and chemotherapy	1.43 (1.09–1.87)	**0.009**		
Surgery and radiation	1.33 (0.96–1.82)	0.081		
Surgery, chemotherapy, and radiation	1.47 (1.15–1.88)	**0.002**		
**How involved is your family doctor/general practitioner/nurse practitioner in your follow-up cancer care (combined categories)?**	-	-		
Not at all involved			Reference	
Do not have a family doctor/GP/nurse/unsure			0.90 (0.67–1.21)	0.500
Somewhat involved			0.91 (0.73–1.12)	0.368
Very involved			0.72 (0.55–0.93)	**0.014**

Bolded *p*-values represent significant (<0.05) variables from the multivariable analysis.

**Table 6:** Multivariable analysis results for the practical domain.

	Moderate or Big Needs		Unmet Needs	
Covariate	Risk Ratio (RR) (95%CI)	*p*-Value	Risk Ratio (RR) (95%CI)	*p*-Value
**Age group (years)**	-	-		
18 to 24			Reference	
25 to 29			1.44 (1.11–1.86)	**0.006**
30 to 34			1.43 (1.11–1.85)	**0.006**
**Gender**				
Male	Reference		-	-
Female	1.46 (1.20–1.76)	**<0.001**		
**Income**				
Less than $25,000	Reference		Reference	
$25,000 to less than $50,000	0.85 (0.65–1.12)	0.261	0.87 (0.67–1.12)	0.298
$50,000 to less than $75,000	0.74 (0.56–0.98)	**0.041**	0.78 (0.60–1.02)	0.071
$75,000 to less than $125,000	0.72 (0.53–0.94)	**0.017**	0.74 (0.58–0.96)	**0.022**
$125,000 or more	0.48 (0.34–0.66)	**<0.001**	0.56 (0.40–0.78)	**<0.001**
Prefer not to answer	0.52 (0.37–0.74)	**<0.001**	0.79 (0.56–1.11)	0.176
**Other chronic condition**			-	-
No	Reference			
Yes	1.22 (1.02–1.47)	**0.028**		
**Treatment received**				
Surgery only	Reference		Reference	
Chemotherapy only	1.61 (1.24–2.08)	**<0.001**	1.30 (1.01–1.66)	**0.037**
Radiation only	1.94 (1.12–3.36)	**0.018**	1.91 (1.15–3.16)	**0.012**
Chemotherapy and radiation	1.80 (1.35–2.40)	**<0.001**	1.33 (1.01–1.75)	**0.041**
Surgery and chemotherapy	1.68 (1.25–2.26)	**<0.001**	0.99 (0.75–1.32)	0.985
Surgery and radiation	1.49 (1.05–2.11)	**0.024**	1.19 (0.84–1.68)	0.332
Surgery, chemotherapy, and radiation	1.76 (1.35–2.31)	**<0.001**	1.03 (0.78–1.35)	0.830

Bolded *p*-values represent significant (<0.05) variables from the multivariable analysis.

## Data Availability

Data is available in a publicly accessible repository that does not issue DOIs. Publicly available datasets were analyzed in this study. This data can be found here: https://www.systemperformance.ca/transitions-study/transition-study-questions/.

## Supplementary Materials

The following are available online at https://www.mdpi.com/article/10.3390/jcm10122628/s1, Table S1: Reported Moderate to Big (MTB) Concerns and Unmet needs among across Physical, Emotional and Practical Domains; Table S2: Univariable analysis results for Moderate To Big (MTB) concerns and Unmet MTB concerns in the Physical, Emotional and Practical domains.
