# Supplementary material for: Biopsychosocial Factors Associated with Supportive Care Needs in Canadian Adolescent and Young Adult Cancer Survivors
Source: J Clin Med. 2021 Jun 15;10(12):2628. doi: 10.3390/jcm10122628 (PMC8232806; doi:10.3390/jcm10122628)
Supplement: Supplementary file 1 [file jcm-10-02628-s001.zip › jcm-1211929-supplementary.pdf]

Supplementary Table S1. Reported Moderate to Big (MTB) Concerns and Unmet needs among across Physical, Emotional and Practical Domains

**Domain**	**No. of Responses**	**MTB Concern Reported *n* (%)**	**Help sought for MTB concern *n* (%)**	**Unmet need reported *n* (%)**
**Physical concerns (*n* = 530)**				
Swelling of arms or legs (lymphedema)	519	52(10.0)	40(76.9)<br>*n* = 52	24(46.1)<br>*n* = 52
Fatigue, tiredness	523	313(59.9)	179(57.2)<br>*n* = 313	202(64.5)<br>*n* = 313
Hormonal, menopause, or fertility	519	198(38.1)	152(76.8)<br>*n* = 198	98(49.5)<br>*n* = 198
Chronic pain or long term pain	519	118(22.7)	93(78.8)<br>*n* = 118	69(58.5)<br>*n* = 118
Bladder and/ or urinary problems (i.e., incontinence)	518	33(6.37)	27(81.8)<br>*n* = 33	16(48.5)<br>*n* = 33
Gastrointestinal problems (digestion issues, bowel, incontinence)	517	105(20.3)	80(76.2)<br>*n* = 105	47(44.8)<br>*n* = 105
Nerve problems (numbness or tingling)	520	129(24.8)	92(71.3)<br>*n* = 129	70(54.3)<br>*n* = 129
Changes to concentration, memory	518	180(34.7)	93(51.7)<br>*n* = 180	135(75.4)<br>*n* = 179
Changes in sexual activity or function	519	138(26.6)	67(48.6)<br>*n* = 138	103(74.6)<br>*n* = 138
**Emotional Concerns (*n* = 530)**				
Depression, sadness, loss of interest in everyday things	506	220(43.5)	123(56.2)<br>*n* = 219	142(64.8)<br>*n* = 219

Anxiety, stress, worry about cancer returning	518	311(60.0)	155(49.8)<br>*n* = 311	207(66.6)<br>*n* = 311
Changes in relationships with family, partners	524	164(31.3)	66(40.2)<br>*n* = 164	124(76.1)<br>*n* = 163
Changes in relationships with friends or coworkers	525	115(21.9)	33(28.7)<br>*n* = 115	96(83.5)<br>*n* = 115
Changes in body image (i.e., confidence in appearance, etc.)	526	241(45.8)	73(30.4)<br>*n* = 240	191(79.6)<br>*n* = 240
Changes in sexual intimacy	521	157(30.1)	55(35.3)<br>*n* = 156	127(81.9)<br>*n* = 155
**Practical Concerns (*n* = 530)**				
Returning to (or future) work or school	522	257(49.2)	114(44.4)<br>*n* = 257	191(74.3)<br>*n* = 257
Getting to and from appointments	521	100(19.2)	54(54.0)<br>*n* = 100	57(57.0)<br>*n* = 100
Taking care of children, elders or other family members	520	60(11.5)	26(44.1)<br>*n* = 59	49(83.1)<br>*n* = 59
Difficulty getting health or life insurance	520	149(28.7)	51(34.2)<br>*n* = 149	135(90.6)<br>*n* = 149
Paying health care bills (e.g.,treatment, services, travel to appointments, assistive devices)	522	109(20.9)	62(56.9)<br>*n* = 109	85(77.9)<br>*n* = 109

Table S2. Univariable analysis results for Moderate To Big (MTB) concerns and Unmet MTB concerns in the Physical, Emotional and Practical domains

Covariate	Physical Domain				Emotional Domain				Practical Domain			
	MTB Concerns		Unmet MTB Concerns		MTB Concerns		Unmet MTB Concerns		MTB Concerns		Unmet MTB Concerns	
	**Risk Ratio (RR) (95%CI)**	***p*-value**	**Risk Ratio (RR) (95%CI)**	***p*-value**	**Risk Ratio (RR) (95%CI)**	***p*-value**	**Risk Ratio (RR) (95%CI)**	***p*-value**	**Risk Ratio (RR) (95%CI)**	***p*-value**	**Risk Ratio (RR) (95%CI)**	**p-value**
**Cancer Type**												
Solid Tumor	Reference											
Hematologic	1.28 (1.08–1.52)	**0.004**	1.19 (0.98–1.45)	0.083	1.23 (1.03–1.47)	**0.02**	1.07 (0.89–1.28)	0.441	1.24 (1.02–1.51)	**0.03**	1.19 (0.99–1.43)	0.060
**Age group (Years)**												
18 to 24	Reference											
25 to 29	1.07 (0.85–1.33)	0.609	1.24 (0.94–1.63)	0.119	1.17 (0.93–1.48)	0.178	0.97 (0.77–1.22)	0.827	1.29 (0.99–1.70)	0.059	1.52 (1.18–1.96)	**0.001**
30 to 34	1.15 (0.93–1.44)	0.196	1.26 (0.96–1.64)	0.092	1.15 (0.91–1.44)	0.248	0.87 (0.69–1.09)	0.234	1.39 (1.06–1.80)	**0.015**	1.45 (1.13–1.87)	**0.004**
**Gender**												
Male	Reference											
Female	1.47 (1.26–1.74)	**<0.001**	1.39 (1.14–1.71)	**0.002**	1.43 (1.20–1.71)	**<0.001**	1.08 (0.91–1.29)	0.362	1.46 (1.20–1.77)	**<0.001**	1.17 (0.98–1.41)	0.102
**Other chronic conditions**												
No	Reference											
Yes	1.14 (0.96–1.35)	0.119	1.04 (0.86–1.27)	0.663	1.33 (1.13–1.57)	**0.001**	1.18 (0.99–1.39)	0.053	1.29 (1.07–1.55)	**0.008**	1.07 (0.88–1.28)	0.442
**Income**												
Less than $25,000	Reference											
$25,000 to less than $50,000	0.85 (0.65–1.09)	0.203	0.84 (0.61–1.14)	0.134	0.84 (0.64–1.10)	0.217	0.82 (0.62–1.07)	0.148	0.81 (0.62–1.07)	0.137	0.94 (0.72–1.22)	0.628

$50,000 to less than $75,000	0.86(0.66–1.11)	0.245	1.04 (0.77–1.40)	0.803	0.94 (0.72–1.22)	0.627	0.86 (0.66–1.12)	0.954	0.70 (0.52–0.93)	**0.014**	0.81 (0.62–1.06)	0.131
$75,000 to less than $125,000	0.71 (0.56–0.92)	**0.008**	0.74 (0.55–1.00)	0.052	0.78 (0.61–1.01)	0.065	0.79 (0.61–1.02)	0.068	0.67 (0.51–0.87)	**0.003**	0.73 (0.57–0.95)	**0.019**
$125,000 or more	0.63 (0.48–0.84)	**0.002**	0.76 (0.54–1.06)	0.107	0.65 (0.48–0.87)	**0.004**	0.69 (0.51–0.93)	**0.016**	0.43 (0.31–0.60)	**<0.001**	0.54 (0.39–0.76)	**<0.001**
Prefer not to answer	0.63(0.47–0.86)	**0.003**	0.75 (0.52–1.07)	0.118	0.65 (0.47–0.89)	**0.008**	0.76 (0.55–1.04)	0.082	0.49 (0.35–0.69)	**<0.001**	0.74 (0.53–1.04)	0.086
**Marital Status**												
Single/Sep/Div/Widowed/prefer not to answer	Reference											
Married or Partnered	1.02 (0.87–1.19)	0.798	1.01 (0.83–1.21)	0.909	0.91 (0.77–1.08)	0.289	0.90 (0.76–1.06)	0.219	1.03 (0.86–1.24)	0.739	1.01 (0.85–1.20)	0.882
**Language (survey completed)**												
French	Reference											
English	1.17 (0.99–1.37)	0.06	1.26 (1.03–1.53)	**0.021**	1.32 (1.11–1.56)	**0.001**	1.17 (0.99–1.39)	0.056	1.01 (0.84–1.22)	0.898	1.03 (0.87–1.23)	0.687
**Education level (highest level)**												
University (Bachelors/Masters or PhD	Reference											
<=High School diploma	1.02 (0.81–1.29)	0.84	0.96 (0.73–1.26)	0.77	0.93 (0.74–1.19)	0.60	1.10 (0.87–1.39)	0.424	1.05 (0.81–1.37)	0.692	1.04 (0.81–1.33)	0.769
<=College	1.29 (1.07–1.56)	**0.007**	1.07 (0.86–1.33)	0.553	1.21 (0.99–1.46)	0.057	1.11 (0.92–1.35)	0.277	1.21 (0.97–1.50)	0.088	1.06 (0.86–1.30)	0.574
Some University	1.22 (0.93–1.58)	0.147	1.06 (0.76–1.47)	0.731	1.02 (0.77–1.36)	0.868	1.08 (0.82–1.44)	0.572	1.25 (0.93–1.69)	0.143	0.99 (0.74–1.33)	0.960
**Population size of geographic location**												
Rural location/Town < 2000 people	Reference											
Town (2,000 to 10,000 people)	0.95 (0.68–1.34)	0.806	0.95 (0.64–1.40)	0.783	0.93 (0.72–1.21)	0.593	0.84 (0.59–1.18)	0.322	0.91 (0.62–1.32)	0.622	0.90 (0.69–1.17)	0.427

Small city (10,000 to 50,000 people)	0.96 (0.72–1.28)	0.780	0.83 (0.59–1.16)	0.267	0.98 (0.73–1.33)	0.915	0.93 (0.69–1.24)	0.621	0.97 (0.70–1.33)	0.837	0.99 (0.69–1.40)	0.941
Large city (>50,000 people)	0.86 (0.67–1.10)	0.234	0.92 (0.71–1.35)	0.783	0.86 (0.67–1.10)	0.234	0.74 (0.57–0.95)	**0.021**	0.78 (0.59–1.03)	0.085	0.91 (0.67–1.23)	0.537
**Current Employment Status**												
Full Time work	Reference											
Part time work	1.23 (0.95–1.59)	0.124	1.09 (0.79–1.49)	0.579	1.35 (1.05–1.74)	**0.021**	1.25 (0.97–1.62)	0.080	1.31 (0.98–1.74)	0.064	1.08 (0.82–1.44)	0.080
Full Time Student	1.11 (0.88–1.39)	0.375	1.02 (0.77–1.35)	0.863	0.98 (0.77–1.26)	0.880	1.04 (0.82–1.33)	0.732	1.02 (0.78–1.34)	0.870	0.88 (0.68–1.15)	0.370
On leave/disability/not working	1.55 (1.28–1.87)	**<0.001**	1.26 (1.01–1.58)	**0.043**	1.39 (1.14–1.69)	**0.001**	1.09 (0.88–1.34)	0.415	1.57 (1.26–1.95)	**<0.001**	1.29 (1.05–1.58)	**0.014**
**How involved is your family doctor/general practitioner/nurse practitioner in your follow–up cancer care?(Combined categories)**												
Not at all involved	Reference											
Do not have a family doc/GP/Nurse/Unsure	0.88 (0.66–1.19)	0.427	1.03 (0.75–1.41)	0.841	0.81 (0.59–1.11)	0.427	0.92 (0.69–1.23)	0.562	0.94 (0.67–1.34)	0.747	0.98 (0.71–1.35)	0.903
Somewhat involved	1.00 (0.81–1.24)	0.964	0.78 (0.62–0.98)	**0.037**	0.99 (0.80–1.23)	0.967	0.91 (0.74–1.12)	0.378	1.11 (0.86–1.42)	0.423	1.04 (0.83–1.31)	0.738
Very involved	0.92 (0.71–1.18)	0.503	0.59 (0.44–0.80)	**0.001**	0.82 (0.63–1.06)	0.136	0.74 (0.57–0.97)	**0.027**	1.09 (0.81–1.46)	0.571	0.87 (0.66–1.14)	0.310
**Physician providing follow-up care**												
No One/Unsure	Reference											
General Practitioner (GP)	0.87 (0.55–1.37)	0.538	0.62 (0.42–0.91)	**0.016**	0.81 (0.54–1.19)	0.289	0.84 (0.58–1.21)	0.366	0.89 (0.57–1.41)	0.633	0.88 (0.59–1.32)	0.542
Oncologist	0.80 (0.55–1.19)	0.273	0.68 (0.42–1.14)	0.118	0.96 (0.60–1.51)	0.849	0.78 (0.50–1.23)	0.290	0.87 (0.51–1.49)	0.624	0.82 (0.50–1.33)	0.416

GP and Oncologist	1.00 (0.67–1.50)	0.989	0.54 (0.36–0.82)	**0.004**	0.94 (0.62–1.42)	0.759	0.84 (0.57–1.23)	0.378	1.05 (0.66–1.68)	0.823	0.77 (0.51–1.17)	0.233
**Treatment received**												
Surgery Only	Reference											
Chemotherapy Only	1.86 (1.48–2.34)	**<0.001**	1.55 (1.18–2.02)	**0.002**	1.58 (1.25–2.00)	**<0.001**	1.25 (0.99–1.57)	0.061	1.58 (1.21–2.07)	**0.001**	1.30 (1.01–1.67)	**0.038**
Radiation only	1.71 (1.01–2.87)	**0.044**	1.32 (0.71–2.48)	0.381	1.37 (0.78–2.41)	0.286	1.47 (0.85–2.52)	0.167	1.82 (1.03–3.23)	0.039	2.06 (1.23–3.44)	**0.006**
Chemotherapy and Radiation	1.67 (1.28–2.18)	**0.0002**	1.27 (0.91–1.76)	0.153	1.19 (0.89–1.59)	0.232	0.94 (0.70–1.27)	0.697	0.94 (0.69–1.30)	0.738	1.33 (1.01–1.76)	**0.042**
Surgery and Chemotherapy	1.80 (1.40–2.31)	0.839	0.98 (0.69–1.36)	0.893	1.30 (0.99–1.71)	0.056	1.03 (0.77–1.36)	0.847	1.51 (1.12–2.04)	0.007	0.99 (0.74–1.32)	0.953
Surgery and Radiation	1.55 (1.13–2.12)	**0.006**	1.18 (0.81–1.74)	0.386	1.43 (1.05–1.96)	**0.025**	1.08 (0.79–1.48)	0.616	1.64 (1.16–2.33)	**0.005**	1.25 (0.88–1.78)	0.206
Surgery, Chemotherapy, and Radiation	2.34 (1.86–2.95)	**<0.001**	1.62 (1.22–2.14)	**0.001**	1.58 (1.23–2.04)	**<0.001**	1.04 (0.80–1.35)	0.759	1.86 (1.41–2.46)	**<0.001**	1.05 (0.80–1.39)	0.692
